# Green Antimicrobials: Innovative Applications of Hops Extracts as Biocontrol Agents

**DOI:** 10.3390/pathogens14050418

**Published:** 2025-04-25

**Authors:** Ana I. Paniagua-García, Ana Ibáñez, Rebeca Díez-Antolínez

**Affiliations:** 1 Centro de I+D de Biocombustibles y Bioproductos del Instituto Tecnológico Agrario de Castilla y León (ITACyL), Villarejo de Órbigo, 24358 Leon, Spain; pangaran@itacyl.es (A.I.P.-G.); dieantre@itacyl.es (R.D.-A.); 2 Instituto Tecnológico Agrario de Castilla y León (ITACyL), Área de Investigación Agrícola, 47071 Valladolid, Spain; 3 Instituto de Investigación de la Viña y el Vino, Escuela de Ingeniería Agraria, Universidad de León, 24009 Leon, Spain

**Keywords:** antibacterial activity, biocontrol, hop extracts, *Humulus lupulus*, natural compounds

## Abstract

Natural compounds represent a fundamental source of antimicrobial agents with applications in numerous industries. This study investigates the antimicrobial properties of different fractions of extracts obtained from six hop varieties, as well as of certain compounds contained in hops and other plants. The results indicate that soft resins exhibit the strongest antibacterial activity among the hop-derived fractions evaluated, reaching a minimum MIC_90_ value of 25 µg/mL (Fuggle variety) against Gram-positive bacteria (*S. aureus*) and 50 µg/mL (Chinook variety) against Gram-negative bacteria (*E. coli*). Furthermore, the composition of hops varies among varieties, resulting in divergent antimicrobial patterns, indicating the necessity for further research to elucidate the origins of these activities. Additionally, while hop-derived fractions exhibited noteworthy antibacterial properties, their antifungal activity against *A. niger* was found to be negligible. In addition, natural compounds such as carvacrol and thymol demonstrated the lowest MIC_90_ values against *E. coli* (130 and 250 µg/mL, respectively) and *S. aureus* (280 and 250 µg/mL, respectively). Moreover, xanthohumol exhibited a better MIC_90_ value against *S. aureus* (3 µg/mL), while no inhibitory effects were observed against *E. coli*. These insights highlight the necessity for further exploration of natural extracts in the development of new antimicrobial agents.

## 1. Introduction

Indubitably, most people associate the antibiotic origin with the serendipitous event of penicillin discovery by Fleming in 1928 [1]. Nevertheless, the formulation of a systematic purification protocol for penicillin, enabling its industrial production, did not materialize until 1944 by Paul Ehrlich, under the auspices of Pfizer [2,3]. This systematic screening approach [3] became the cornerstone of drug search strategies in the pharmaceutical industry, resulting in thousands of drugs identified and translated into clinical practice, such as the sulfonamidochrysoidine (KI-730, Prontosil) [4]. However, it did not take long for evidence that some microbial species could destroy antimicrobial compounds through enzymatic degradation [5]. For instance, some members of the Gram-negative *Enterobacteriaceae* group have developed resistance not only to the original penicillin but also to semi-synthetic penicillins, cephalosporins, and carbapenems [6]. Mortality rates due to multidrug-resistant bacterial (MRB) infections are high. Every year, approximately 25,000 patients in the EU die from infections caused by MRB, and more than 63,000 patients in the United States succumb to hospital-acquired bacterial infections [7]. One popular approach to combat these new MRB is the search for novel antimicrobial compounds from new natural sources, primarily focused on plants and microorganisms.

The hop plant (*Humulus lupulus* L.) is a perennial dioecious plant belonging to the Cannabaceae family, widely distributed throughout the Northern Hemisphere (i.e., Europe, Asia, and North America) [8,9]. Hops have been cultivated since ancient times, primarily for the brewing industry, especially in countries such as Germany and the USA [10]. It is exclusively the female plants that produce the inflorescences, which house the lupulin glands [11]. These glands contain high-value compounds that may be concentrated in the essential oils and resins after an extraction process [12]. The essential oil represents 0.3 to 3% (*v*/*w*) of the whole hop strobile weight, and the main constituents of these essential oils are terpenes (like β-myrcene and β-farnesene), aldehydes, ketones, carboxylic acids, and esters, although the compound profile might vary significantly depending on the hop variety [13,14].

Total hop resins are composed of soft and hard resins, which can be separated based on their solubility in different solvents [15,16]. Soft resins, soluble in hexane, consist of bitter acids including humulone, cohumulone, and adhumulone (α-acids), as well as lupulone, colupulone, and adlupulone (β-acids) [16]. These compounds are responsible for the characteristic bitterness, stability, and froth of beer [17]. Additionally, humulone and lupulone exhibit a broad spectrum of antimicrobial activity against both Gram-positive and Gram-negative bacteria, as well as some actinomycetes, yeasts, and fungi [18]. In fact, the antibacterial properties of hops are often attributed to these bitter acids, which have also been reported to display antiviral activity, including against viruses such as Influenza A (H3N2) [14,19]. In contrast, hard resins are the hexane-insoluble fraction of total resins, which are soluble in methanol, ethanol, and diethyl ether [15]. The primary compound found in hard resins is xanthohumol, a prenylated flavonoid that is unique to hop inflorescences [16]. Xanthohumol holds significant clinical importance due to its antioxidant, anti-inflammatory, antiplasmodial, anti-obesity, and anticancer activities [20]. Moreover, xanthohumol has demonstrated considerable potential in the prevention and treatment of various human cancers, including breast, colon, and ovarian [8,9], as well as antimicrobial, antiviral, and antifungal activities [20]. On the other hand, both lupulone and xanthohumol have a positive synergic action in inhibiting bacterial growth when added with polymyxin B sulfate, tobramycin, and ciprofloxacin [21].

In addition to the valuable compounds present in hop resins and essential oils, phenolic compounds (in particular prenylatedacylphloroglucinols and prenylated flavonoids) represent another type of compounds of great industrial interest [12,14]. A variety of hop phenolic compounds have been identified, including flavonol derivatives (e.g., quercetin and kaempferol glycosides), flavan-3-ols (e.g., catechin, epicatechin, tannins and proanthocyanidins), phenolic carboxylic acids (e.g., chlorogenic acid and its isomers, coumaroylquinic acids and feruloylquinic acids) and other polyphenols (e.g., prenylflavonoids, resveratrol, multidifols) [15,16,22]. The concentration of these secondary metabolites progressively rises throughout the development of female hop cones, and this accumulation is influenced by various factors, including the specific cultivar and prevailing climatic conditions [14].

Previous analysis led to the development of a sequential extraction method to recover the high-value compounds from different fractions of hop extraction [15]. This approach led to the successful recovery of the highest levels of bitter acids in soft resins, xanthohumol in hard resins, and phenolic compounds in spent solids. Furthermore, some fractions exhibited high antioxidant activity, particularly the soft resins, suggesting the possibility that they may also exhibit antimicrobial properties. However, the biological activities of these complex extracts remain largely underexplored compared to individual purified compounds.

In this study, we systematically assess the antimicrobial potential of fractionated extracts obtained from six hop varieties, as well as 21 pure plant-derived compounds. Given the chemical variability between hop varieties, we hypothesize that differences in extract composition should translate into differences in antimicrobial efficacy. Therefore, determining which hop varieties yield the most active fractions may help identify candidates of particular interest for the eco-sustainable production of new biocidal agents. To our knowledge, this is the first study combining varietal comparison, sequential extraction, and broad-spectrum antimicrobial screening against both Gram-positive and Gram-negative bacteria, as well as fungi.

## 2. Materials and Methods

### 2.1. Natural Compounds and Biomasses

Eleven natural compounds from plant origin (e.g., vanillin, thymol, carvacrol, eugenol and curcumine), some of which may be found in hop (including xanthohumol, myricetin, myrcene, β-caryophyllene, β-farnesene and humulene), along with ten natural phenolic acids (gallic acid, caffeic acid, vanillic acid, ferulic acid, *p*-coumaric acid, 3-hydroxybenzoic acid, 4-hydroxybenzoic acid, 3,4-dihydroxybenzoic acid, syringic acid, and chlorogenic acid), all provided by Sigma-Aldrich (Steinheim, Germany), were analyzed to determine their antimicrobial activities. The selection of these compounds was based on their natural occurrence in diverse plant species and the feasibility of their recovery through techniques such as extraction, steam distillation, and physico-chemical pretreatments.

Similarly, different fractions obtained from six hop varieties (Nugget, Cascade, Columbus, Fuggle, Magnum, and Chinook), kindly supplied in pellet form by Órbigo Valley S.L. (Villamor de Órbigo, León, Spain), were analyzed to determine their antimicrobial activities against both bacterial and fungal strains.

As detailed by Paniagua-García et al. [15], each hop variety was subjected to two different treatments to obtain five fractions (essential oils, total, soft and hard resins, and spent solids). The essential oils were extracted through steam distillation, using a Clevenger distillation apparatus, in accordance with the European Brewery Convention (EBC) method 7.10 (https://brewup.eu/ebc-analytica/hops-and-hop-products/hop-oil-content-of-hops-and-hop-products/7.10, accessed on 20 September 2023). The main compounds of hop essential oils are described in Table 1.

**Figure 1:**
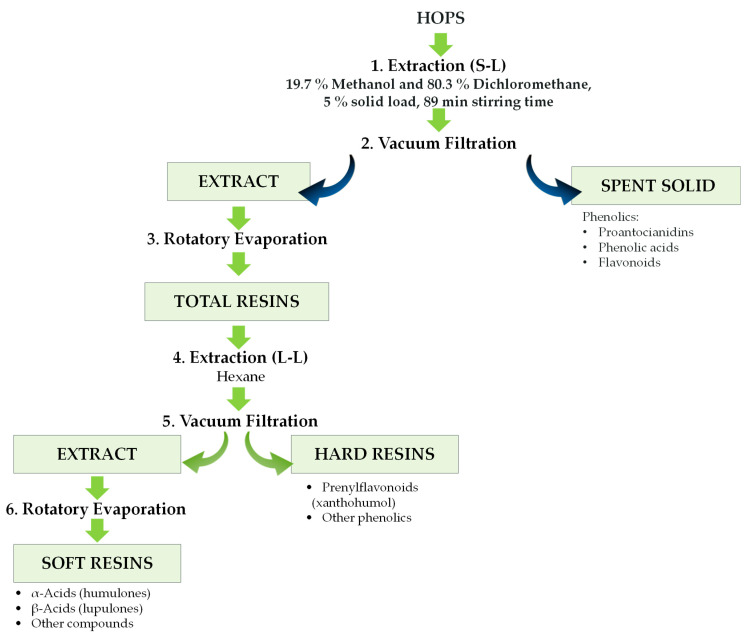
Flow diagram of the optimized sequential extraction procedure from hops.

In order to obtain the different types of resins (total, soft, and hard resins) from hops, as well as the spent solids, a process for the extraction and fractionation of their high-value compounds was developed and optimized by Paniagua-García et al. [15]. The optimized sequential extraction procedure (Figure 1) enabled the attainment of maximum recoveries of α-acids and β-acids in soft resins, xanthohumol in hard resins, and phenolics in spent solids. The chemical composition of the four aforementioned fractions, for the six hop varieties, is summarized in Table 2.

### 2.2. Microbial Cultures and Growth Conditions

To determine the antimicrobial effect of both the natural compounds and the hop extract fractions, one Gram-negative (*Escherichia coli* CECT 515) and one Gram-positive (*Staphylococcus aureus* CECT 239) bacterial species, both from Colección Española de Cultivos Tipo, Valencia, Spain, as well as one fungal specie (*Aspergillus niger* DSM 1957) from DSMZ (Deutsche Sammlung von Mikroorganismen und Zellkulturen Germany), were used.

### 2.3. Preparation of Antimicrobial Solutions

In consideration of the hydrophobic nature of the compounds, stock solutions of natural compounds were prepared in Mueller Hinton medium alone or with the addition of 10% (*v*/*v*) ethanol or 2% (*v*/*v*) Tween 80. Therefore, to obtain stock solutions, solubility tests were previously performed for each compound using the three media described above, and the solvent that provided the maximum amount of dissolved compound was selected. In addition, the concentration of ethanol and Tween 80 is twice the maximum concentration that exhibits no antimicrobial activity against the microorganisms tested. Since the experiments involve at least a 1:2 dilution of the stock solution, both ethanol and Tween 80 remain at a maximum concentration of 5% and 1%, respectively. The stock solutions prepared at the maximum soluble concentration of each natural compound are detailed in Table 3.

Regarding the stock solutions of the essential oils and the different fractions obtained from hop extraction, they were also prepared in Mueller Hinton medium supplemented with 10% (*v*/*v*) ethanol, at a concentration of 1600 µg/mL. Additionally, an extraction of the spent solids was performed. For this purpose, 1.25 g of solids were extracted with 50 mL of boiling water with a reflux condenser for 20 min.

### 2.4. Minimum Inhibitory Concentration (MIC)

Minimum Inhibitory Concentration (MIC) is defined as the lowest concentration of a natural compound that inhibits the growth of the tested strain at the specified percentage. Thus, MIC_50_ and MIC_90_ are defined as the concentrations at which 50% or 90% of bacterial growth are inhibited, respectively [23].

MIC determinations were conducted in 96-well microplates with a final volume of 200 µL, utilizing a SpectroStar Nano plate Microplate Reader (BMG LABTECH, Ortenberg, Germany). The bacterial inoculum underwent cultivation at 37 °C and 150 rpm for 18–20 h, followed by subsequent dilution to an OD 600 nm of 0.1 (approximately 1 × 10^6^ cells/mL). Serial 1:2 dilutions of all compounds were prepared from stock solutions, with each dilution receiving 100 µL of the bacterial inoculum. The dilution process consistently employed the same solvent as utilized in the respective stock solutions, with the solvent itself employed as a blank in the measurements. For the positive control, 100 µL of inoculum and 100 µL of solvent were utilized, while the negative control comprised 100 µL of the stock solution and 100 µL of solvent. Each MIC determination was made in triplicate.

### 2.5. Minimum Bactericidal Concentration (MBC)

Minimum Bactericidal Concentration (MBC) is defined as the lowest concentration of the antimicrobial compound required to kill 99%, 99.9%, or 99.99% (MBC_99_, MBC_99.9_, or MBC_99.99_, respectively) of the viable cells in the MIC inoculum [24].

A 100 μL aliquot was transferred from the wells with no evidence of growth during MIC analysis to the surface of the Müller Hinton agar plates. The plates were incubated at 37 °C for 24 h. MBC test was performed in triplicate with both bacterial strains and all the natural compounds. When the lowest concentration aliquot from the MIC resulted in an MBC_99.99_, the lower values of MBC were not determined. Each MBC determination was made in triplicate.

### 2.6. Antifungal Activity Determination

To determine the antifungal activity of the natural compounds, the Kirby–Bauer disc diffusion method was used. An *A. niger* inoculum was incubated at 25 °C for 72 h and subsequently diluted to an adjusted absorbance of 0.1. Mueller Hinton agar plates were inoculated with 100 µL of the inoculum, and 10 µL of each stock solution was applied using the diffusion discs. The plates were then incubated at 25 °C for 72 h, after which the diameter of the inhibition zone was measured.

## 3. Results

### 3.1. Minimum Inhibitory Concentration (MIC) of Natural Compounds

The comparative analysis of the MIC of natural compounds against the Gram-negative *E. coli* and the Gram-positive *S. aureus* unveils distinct patterns of antimicrobial efficacy. Particularly, a pronounced effectiveness is observed against *E. coli* in comparison to *S. aureus*, requiring higher concentrations of certain compounds such as carvacrol, eugenol, syringic acid, vanillic acid, and vanillin to achieve equivalent inhibitory effects (Table 4).

It is important to note that carvacrol and thymol are the most potent antimicrobial agents against both bacterial strains under analysis. These agents show MIC_90_ values of 130 and 250 µg/mL for *E. coli* and 280 and 250 µg/mL for *S. aureus*, respectively. Conversely, compounds characterized by their strong hydrophobic nature, such as curcumine, myricetin, and xanthohumol, were evaluated at substantially diminished concentrations, precluding precise determination of MIC values. Additionally, other compounds such as β-farnesene, humulene, myrcene, and β-caryophyllene could be evaluated at higher concentrations. However, these compounds did not demonstrate any inhibitory effect against the two bacteria analyzed. Notably, while the concentrations tested for xanthohumol and gallic acid were insufficient to elicit an effect on *E. coli*, significant antimicrobial activity against *S. aureus* was demonstrated (MIC_90_ values of 3 and 6000 µg/mL, respectively). These findings underscore the differential susceptibility of *E. coli* and *S. aureus* to different natural compounds.

### 3.2. Minimum Inhibitory Concentration (MIC) of Hop Extracts

In contrast to the analysis of natural compounds, it is evident that different hop extract fractions exhibit a greater antimicrobial effect against *S. aureus* compared to *E. coli*. In numerous instances, particularly with essential oils and spent solids, the MIC values for *E. coli* could not be determined as they surpassed the maximum concentration tested (Table 5).

Among the analyzed hop extract fractions, soft resins emerge as the most potent antimicrobial agent against both *E. coli* and *S. aureus*, reaching MIC_90_ values ranging from 50 to 400 µg/mL for *E. coli* and from 25 to 100 µg/mL for *S. aureus*, depending on hop variety. Conversely, essential oils, hard resins, and total resins exhibited inhibitory effects only against *S. aureus*, with MIC_90_ values ranging from 30 to 115 µg/mL, 25 to 100 µg/mL, and 100 to 200 µg/mL, respectively. In the case of spent solids, no inhibitory effects were detected against either bacterium in the range of concentrations analyzed.

These observations underscore the differential antimicrobial responses of *E. coli* and *S. aureus* to hop extract fractions, with soft resins demonstrating notable efficacy across both bacterial species. It is also noteworthy that soft resins extracted from the Chinook variety demonstrated the greatest efficiency against *E. coli* (50 µg/mL of MIC_90_), and soft resins from the Fuggle variety demonstrated the greatest efficiency against *S. aureus* (25 µg/mL of MIC_90_).

### 3.3. Minimum Bactericidal Concentration (MBC) of Natural Compounds

Based on the MIC results, the MBCs at 99%, 99.9%, and 99.99% were determined. Table 6 presents the data for the natural compounds used in the analysis for the two analyzed strains (*E. coli* and *S. aureus*).

It is crucial to emphasize that, for each bacterium, once the highest percentage of the bactericidal effect of a natural compound could be determined within the range of concentrations analyzed, lower percentages were not assessed.

Consistent with the MIC analysis, natural compounds exert greater bactericidal activity against *E. coli* compared to *S. aureus*, with the latter requiring higher quantities to achieve similar (or even inferior) effects. This trend persists, with the exception of xanthohumol and gallic acid, which again demonstrated greater activity against *S. aureus*. Furthermore, the highest bactericidal effect against *E. coli* was exhibited by carvacrol, thymol, and eugenol (MBC_99.99_ of 125, 252, and 500 µg/mL, respectively). In the same way, the highest bactericidal effect against *S. aureus* was shown by ferulic acid and vanillic acid (MBC_99.99_ of 2500 µg/mL), followed by 3,4-dihydroxybenzoic acid, 3-hydroxybenzoic acid, and 4-hydroxybenzoic acid (MBC_99.99_ of 3000 µg/mL). Conversely, chlorogenic acid, curcumine, β-farnesene, humulene, myrcene, and myricetin did not demonstrate bactericidal activity against either of the bacteria within the range of concentrations studied.

### 3.4. Minimum Bactericidal Concentration (MBC) of Hop Extracts

As was the case with natural compounds, the bactericidal effect of a hop-derived fraction was evaluated for each bacterium. Once the highest percentage of this effect could be ascertained within the range of concentrations that were analyzed, percentages lower than that were not assessed.

Interestingly, mirroring the MIC analyses of hop extracts, a greater bactericidal effect against *S. aureus* is observed. Therefore, soft resins, hard resins, and total resins exhibited the best bactericidal effects against *S. aureus*. Furthermore, the Columbus variety was the most effective in achieving the highest effects (MBC_99.99_) with lower concentrations (100 µg/mL for soft resins, 400 µg/mL for hard resins, and 200 µg/mL for total resins). Conversely, no bactericidal effect of any hop extract fraction was observed against *E. coli*. For this reason, Table 7 shows only the bactericidal effects against *S. aureus*.

### 3.5. Antifungal Effect of Both Natural Compounds and Hop Extracts

Regarding the antifungal activity of the natural compounds under investigation, it is noteworthy that only pure thymol exhibited inhibitory effects on the growth of *A. niger*. Conversely, none of the fractions of hop extracts demonstrated antifungal activity.

## 4. Discussion

Throughout history, natural compounds have been extensively applied in food preservation practices, which, albeit unknowingly at the time, primarily aimed at preventing or controlling the growth of some microorganisms. Among the most recognized methods are salting and acidification, although traditionally, other natural compounds such as spices (oregano, cinnamon, cloves, black pepper, or basil, among others) and organic acids (such as acetic acid or citric acid) have been employed [25,26]. Building on these foundations, the current quest for novel antimicrobial compounds once again features natural compounds (mainly of plant, animal, or microbial origin) [27,28].

Hop plants are distinguished by their perennial roots (rhizomes), green leaves with three lobes, and female inflorescences, also known as cones, strobiles, or hops. While hop plants have traditionally been used in the brewing industry, in recent years, they have also been cultivated for pharmaceutical purposes, since hop has been associated with the treatment of anxiety and insomnia, the alleviation of menopausal symptoms, the management of digestive disorders, and even the potential purification of antimicrobial compounds [14].

Back in the 1990s, antimicrobial activity assays using hop essential oils were already being conducted [29], revealing intense activity against Gram-positive bacteria, but not against Gram-negative ones. Consistently, all the varieties analyzed in this study (Nugget, Cascade, Columbus, Fuggle, Magnum, and Chinook) exhibited antibacterial effects against *S. aureus* but not against *E. coli*, reinforcing results reported by other researchers. To our knowledge, only one study has reported, while rare, slight-to-moderate activity of hops essential oil against some Gram-negative bacteria such as *E. coli* or *Yersinia enterocolitica* [14]. Theoretically, differences may be observed in the antibacterial properties of the essential oils from different hop varieties. The antimicrobial activities of essential oils are highly associated with their chemical constituents, which, in turn, are largely influenced by factors such as hop varieties and extraction methods [15]. Interestingly, in our case, the antimicrobial activity pattern persists across most of the hop varieties analyzed, in all tested hop extract fractions, with few exceptions, such as the Nugget variety in essential oils antimicrobial analysis. In general, monoterpenes and terpenoids dominate the chemical constituents of hop essential oils, including myrcene, humulene, and caryophyllene [30,31,32]. However, these compounds do not show antibacterial activity against either *S. aureus* or *E. coli* at the concentrations analyzed, suggesting that the observed activity in the essential oils may be due to the presence of other minor compounds, not yet reported. For example, β-farnesene is a minor compound that can be synthesized in certain hop varieties [33], although β-farnesene showed no antibacterial activity at the concentrations analyzed. Our results showed that the essential oils extracted from the Cascade variety, with the highest content of geraniol (1.64%) and β-farnesene (7.96%) [15], achieved the best values of *S. aureus* growth inhibition (MIC_90_ of 30 µg/mL). In addition, the Nugget variety exhibited significantly lower antibacterial activity compared to the other varieties. If we consider their composition, as shown in Table 1 of the study by Paniagua-García [15], the content of β-pinene (0.16%) and geraniol (0.08%) in the essential oils from the Nugget variety are lower than in the other hop varieties. These results are in agreement with previous works that reported high antimicrobial activity of both compounds against Gram-positive bacteria such as *S. aureus* [34,35,36,37,38].

On the other hand, we observed that all fractions, except spent solids, exhibited antibacterial activity with variable results among the hop varieties tested. In this case, the fraction with the highest antibacterial activity is not the essential oils, but rather the soft resins. In fact, it has been reported that extracts rich in essential oils are less active compared to extracts rich in prenylated acylphloroglucinols such as humulones and lupulones [14]. This suggests that, despite essential oils being the most studied fraction, other fractions may be more promising for the development of novel antimicrobial agents.

The total resins of the hop cones are divided into soft and hard resins. The major compounds contained in soft resins are humulone (α-acids) and lupulone (β-acids) [17]. Both compounds interfere with the phosphoenolpyruvate (PEP) system of Gram-positive bacteria, resulting in membrane leakage and a subsequent inhibition of respiration and synthesis of proteins, DNA, and RNA. However, Gram-negative bacteria may not be affected, most likely due to the serum phosphatides present in the phospholipids containing outer membrane [17,39]. Once again, comparing the results of the antimicrobial analysis with the composition of the fractions obtained for the different hop varieties [15], it can be observed that, in general, the soft resins fraction contains the highest content of α-acids (26.54–43.22 g/100 g) and β-acids (13.31–29.9 g/100 g), compared to 5.54–12.39 g/100 g and 0.89–2.04 g/100 g, respectively, in hard resins. Nonetheless, the Chinook variety did not conform to the antimicrobial activity pattern (Table 5), as soft resins exhibited significant activity against *E. coli*, while requiring higher concentrations than other varieties to act against *S. aureus*. The Chinook variety contains higher quantities of cohumulone (13.03 ± 0.90 g/100 g) and lesser of adlupulone (5.89 ± 0.23 g/100 g) in the soft resins compared to other varieties, and, although it presents higher concentrations of α-acids (43.22 ± 2.84 g/100 g) than the rest, albeit lower quantities of β-acids (13.31 ± 0.54 g/100 g) [15]. Interestingly, it has been reported that β-acids precisely exhibit greater antimicrobial activity than α-acids [40], so these differences in the composition of the soft resins may account for the observed variations in the antibacterial activity of the extracts.

The total resins of the hop cones are divided into soft and hard resins. The major compounds contained in soft resins are humulone (α-acids) and lupulone (β-acids) [17]. Both compounds interfere with the phosphoenolpyruvate (PEP) system of Gram-positive bacteria, resulting in membrane leakage and a subsequent inhibition of respiration and synthesis of proteins, DNA, and RNA. However, Gram-negative bacteria may not be affected, most likely due to the serum phosphatides present in the phospholipids containing outer membrane [17,39]. Once again, comparing the results of the antimicrobial analysis with the composition of the fractions obtained for the different hop varieties [15], it can be observed that, in general, the soft resins fraction contains the highest content of α-acids (26.54–43.22 g/100 g) and β-acids (13.31–29.9 g/100 g), compared to 5.54–12.39 g/100 g and 0.89–2.04 g/100 g, respectively, in hard resins. Nonetheless, the Chinook variety did not conform to the antimicrobial activity pattern (Table 5), as soft resins exhibited significant activity against *E. coli*, while requiring higher concentrations than other varieties to act against *S. aureus*. The Chinook variety contains higher quantities of cohumulone (13.03 ± 0.90 g/100 g) and lesser of adlupulone (5.89 ± 0.23 g/100 g) in the soft resins compared to other varieties, and, although it presents higher concentrations of α-acids (43.22 ± 2.84 g/100 g) than the rest, albeit lower quantities of β-acids (13.31 ± 0.54 g/100 g) [15]. Interestingly, it has been reported that β-acids precisely exhibit greater antimicrobial activity than α-acids [40], so these differences in the composition of the soft resins may account for the observed variations in the antibacterial activity of the extracts.

Conversely, hard resins constitute the fraction of the total resin soluble in methanol and diethyl ether. Among the constituents of these hard resins is the renowned prenylflavonoid xanthohumol [17], which may be responsible for the observed activity. Both the results obtained in the bioassay with the hard resins and those conducted with commercial xanthohumol reveal greater antimicrobial activity against *S. aureus* than against *E. coli*. Xanthohumol has demonstrated potent activity against Gram-positive bacteria such as *S. aureus*, although its effect on Gram-negative bacteria is more specific, acting exclusively on certain species [17,21]. Mechanisms underlying their antimicrobial activity have not been extensively studied, but some reports suggest that it can affect bacterial cell membrane integrity, interfering with fatty acid metabolism and leading to an accumulation of protons intracellularly, with the subsequent cell starvation [41]. In this case, xanthohumol may be responsible for the different patterns described by the Magnum and Chinook varieties, which show no activity against *E. coli*. When analyzing the composition of the hard resins of the different varieties [15], both hop varieties have the highest values of xanthohumol among those analyzed (9.13 and 7.36 g/100 g, respectively). However, no compound is observed in the rest of the varieties that could be responsible for the activity against *E. coli*.

Despite the notable antibacterial properties exhibited by hop extracts, their antifungal activity against *A. niger* is negligible. However, previous reports have documented antifungal activity of certain hop extracts against agriculturally significant fungi. One such example is the potent antifungal activity of isoxanthohumol, the major prenylated flavonoid found in hop extracts, against *Botrytis cinerea*. It has been reported that isoxanthohumol interferes with the entire metabolic pathway of fungi, including the tricarboxylic acid cycle [42]. Similarly, nanoemulsions of essential oils from hop extracts have been demonstrated to inhibit the mycelial growth and spore germination of *Fusarium graminearum* by altering the total lipid and chitin content in the outer cell membrane, as well as impairing cytoplasmic membrane permeability [31]. Additionally, both the crude extract and the essential oil from hop significantly reduced the growth of *Zymoseptoria tritici*, the most frequently occurring and damaging pathogen in wheat crops [43]. Thus, despite the antifungal properties of different fractions of hop extracts having been reported, their mechanisms of action have not been fully elucidated, but it appears that the activity is more species-specific.

These findings underscore the significant potential of natural extracts as antimicrobial agents. The results of our study demonstrated that various natural compounds, such as carvacrol, thymol, and eugenol, exhibit substantial antimicrobial activity, with MIC_90_ values at concentrations below 500 µg/mL. These low MIC_90_ values indicate that even minimal amounts of these compounds can effectively inhibit microbial growth. For instance, vanillin has been extensively studied for its antimicrobial properties. As a standalone agent, vanillin has been shown to possess strong antimicrobial effects [44,45], but when combined with chitosan, an enhancement of these effects has been observed [46,47], making it a valuable compound for use in several industries. This combination has proven particularly effective, suggesting that the integration of two or more natural compounds, either among themselves or with other substances, can lead to improved antimicrobial strategies. Such is the case with the synergistic combination of thymol and carvacrol, which has been shown to significantly amplify antimicrobial efficacy. Studies have demonstrated that when used together, these compounds work more effectively than when used individually, providing a powerful means of combating microbial infections [48]. This synergy suggests that exploring combinations of natural compounds could yield potent antimicrobial formulations that leverage the strengths of each component.

Last, but not least, phenolic compounds represent one of the most diverse groups of secondary metabolites in edible plants. Among them, phenolic acids are small molecules characterized by a carboxylic acid group on their non-active end, which exhibit broad antimicrobial properties. These properties are mainly attributed to their ability to destabilize the bacterial cytoplasmic membrane, alter membrane permeability, inhibit extracellular microbial enzymes, directly disrupt microbial metabolism, and deprive microbes of essential substrates for growth [49]. Additionally, phenolic acids can alter bacterial polarity by modifying surface electron acceptors in both Gram-positive (increasing acceptor components) and Gram-negative (decreasing acceptor components) strains, with increased concentrations leading to significant cell membrane damage [50]. Indeed, certain phenolic acids have demonstrated potent antimicrobial activity against *E. coli*, as well as some *Lactobacillus* and *Staphylococcus* strains [51,52]. In our study, all analyzed phenolic acids showed antibacterial activity against both *E. coli* and *S. aureus*, although no antifungal activity was observed.

As a result, these findings highlight the significant potential of these natural extracts as a foundation for developing new antimicrobial agents, emphasizing their value in addressing the increasing challenge of antibiotic resistance. Although these compounds cannot match the efficacy of commercial antibiotics, direct comparisons are impractical given that the concentration required to achieve comparable effects would be unrealistically high. Nevertheless, the alarming rise of MRB necessitates the exploration of new candidates, such as these natural extracts, to serve as a starting point for the development of innovative pharmaceuticals capable of addressing this critical global health issue. Future research will focus on identifying the chemical composition of these fractions to isolate and purify key compounds, enabling more practical and targeted applications of hop extracts in the development of antimicrobial solutions. The results of this study could serve as a foundation for the expansion of hop-derived fraction production on an industrial scale, paving the way for the development of new applications beyond the traditional brewing industry.

## 5. Conclusions

This study underscores the significant potential of hop extract fractions, particularly soft resins, as promising antimicrobial agents. The findings indicate that soft resins exhibit the highest antibacterial activity against both *S. aureus* and *E. coli*, positioning them as promising candidates for further research in the quest for new antimicrobial compounds. However, other fractions, such as essential oils and spent solids, showed minimal activity against *E. coli*, suggesting they may be effective sources of antibacterial compounds exclusively against Gram-positive bacteria.

Moreover, the observed variation in antimicrobial activity among different hop varieties suggests that the specific composition of these varieties plays a crucial role, warranting further investigation. Despite the strong antibacterial properties, the antifungal activity of hop extracts against *A. niger* was negligible. Nevertheless, the substantial antimicrobial activity exhibited by compounds like carvacrol, thymol, and eugenol at low concentrations highlights the potential of natural extracts in developing new antimicrobial agents.

These results advocate for the continued exploration and utilization of natural compounds, particularly from hop extracts, to address the growing challenge of antibiotic resistance. Furthermore, future research should focus on isolating the active compounds from hop fractions, particularly from the soft resins, to better understand their mechanisms of action. In vivo studies will also be crucial to confirm the efficacy and safety of these compounds in real-world applications. By pursuing these avenues, we can enhance the development of novel, eco-sustainable antimicrobial agents and apply them to different industries.

## Figures and Tables

**Table 1:** Essential oil compounds profile (%, rel) of the six hop varieties.

Hop Variety	Nugget	Columbus	Chinook	Magnum	Cascade	Fuggle
β-Pinene (%, rel)	0.16	0.66	0.23	0.37	0.40	0.25
Myrcene (%, rel)	51.84	56.43	29.70	64.00	44.18	42.30
Limonene (%, rel)	0.66	0.66	0.50	0.84	0.61	0.61
Linalool (%, rel)	1.15	0.77	0.59	0.63	0.61	0.75
Geraniol (%, rel)	0.08	0.75	0.51	0.62	1.64	0.27
2-Undecanone (%, rel)	0.52	0.19	0.32	0.62	0,24	0.49
β-Cariophyllene (%, rel)	9.01	7.69	10.39	5.96	7.34	9.75
β-Farnesene (%, rel)	ND	ND	ND	ND	7.96	4.37
Humulene (%, rel)	19.84	15.03	24.42	14.29	18.16	25.74

%, rel: percentage of relative area; ND: not detected.

**Table 2:** Weight and chemical composition [α-acids, β-acids, xanthohumol, and total phenolic compounds (TPC)] of the initial hop pellets and the fractions obtained after the sequential extraction process of the six hop varieties. Reproduced from Paniagua-García et al. [15].

	Weight (g)	α-Acids (g/100 g)	β-Acids (g/100 g)	Xanthohumol (g/100 g)	TPC (g GAE/100 g)
Nugget					
Initial Hops	20.00	10.26 ± 0.12	3.65 ± 0.04	0.68 ± 0.01	2.10 ± 0.02
Soft Resins	4.15 ± 0.18	39.19 ± 1.03	15.05 ± 0.58	0.21 ± 0.00	NA
Hard Resins	1.96 ± 0.07	6.94 ± 0.40	0.89 ± 0.05	5.56 ± 0.25	3.07 ± 0.15
Spent Solid	14.33 ± 0.08	0.36 ± 0.13	0.06 ± 0.02	0.02 ± 0.01	1.97 ± 0.04
Columbus					
Initial Hops	20.00	11.96 ± 0.13	4.13 ± 0.02	0.70 ± 0.01	1.75 ± 0.03
Soft Resins	3.92 ± 0.22	38.37 ± 1.47	15.29 ± 0.54	0.23 ± 0.00	NA
Hard Resins	1.64 ± 0.07	12.39 ± 1.21	1.45 ± 0.30	5.61 ± 0.14	3.31 ± 0.23
Spent Solid	14.57 ± 0.06	1.45 ± 0.08	0.43 ± 0.03	0.08 ± 0.01	1.75 ± 0.04
Chinook					
Initial Hops	20.00	9.02 ± 0.29	2.58 ± 0.09	0.55 ± 0.01	2.75 ± 0.03
Soft Resins	3.30 ± 0.13	43.22 ± 2.84	13.31 ± 0.54	0.36 ± 0.03	NA
Hard Resins	1.19 ± 0.42	5.54 ± 0.93	0.98 ± 0.04	7.36 ± 2.93	4.29 ± 0.68
Spent Solid	15.03 ± 0.25	0.37 ± 0.06	0.07 ± 0.02	0.02 ± 0.00	2.94 ± 0.09
Magnum					
Initial Hops	20.00	6.84 ± 0.08	2.62 ± 0.03	0.53 ± 0.01	2.81 ± 0.02
Soft Resins	2.76 ± 0.05	41.89 ± 0.64	17.01 ± 0.31	0.67 ± 0.01	NA
Hard Resins	0.85 ± 0.07	6.96 ± 0.86	1.68 ± 0.10	9.13 ± 0.74	5.24 ± 0.49
Spent Solid	15.73 ± 0.06	0.23 ± 0.05	0.05 ± 0.02	0.02 ± 0.00	2.78 ± 0.07
Cascade					
Initial Hops	20.00	4.68 ± 0.01	4.58 ± 0.04	0.33 ± 0.00	2.75 ± 0.01
Soft Resins	2.76 ± 0.02	26.54 ± 0.38	29.29 ± 0.78	0.51 ± 0.02	NA
Hard Resins	1.10 ± 0.20	6.79 ± 0.49	1.98 ± 0.48	4.28 ± 0.88	5.93 ± 0.68
Spent Solid	15.77 ± 0.12	0.19 ± 0.02	0.08 ± 0.01	0.01 ± 0.00	2.74 ± 0.03
Fuggle					
Initial Hops	20.00	6.30 ± 0.10	2.99 ± 0.03	0.40 ± 0.00	3.28 ± 0.03
Soft Resins	3.30 ± 0.54	30.42 ± 3.71	16.15 ± 1.98	0.57 ± 0.11	NA
Hard Resins	1.01 ± 0.13	8.62 ± 2.63	2.04 ± 0.81	5.71 ± 0.82	4.73 ± 0.71
Spent Solid	15.97 ± 0.15	0.19 ± 0.04	0.05 ± 0.01	0.01 ± 0.00	3.56 ± 0.17

GAE: gallic acid equivalents; NA: not analyzed.

**Table 3:** Composition of the stock solutions of the natural compounds.

Natural Compound	Solvent	Concentration (µg/mL)
3,4-Dihydroxybenzoic acid	Mueller Hinton Medium	12,000
3-Hydroxybenzoic acid	Mueller Hinton Medium + 10% (*v*/*v*) Ethanol	6000
4-Hydroxybenzoic acid	Mueller Hinton Medium + 10% (*v*/*v*) Ethanol	6000
Caffeic acid	Mueller Hinton Medium + 2% (*v*/*v*) Tween 80	10,000
Carvacrol	Mueller Hinton Medium	4000
Chlorogenic acid	Mueller Hinton Medium + 2% (*v*/*v*) Tween 80	5000
Curcumine	Mueller Hinton Medium + 10% (*v*/*v*) Ethanol	50
Eugenol	Mueller Hinton Medium + 10% (*v*/*v*) Ethanol	2000
β-Farnesene	Mueller Hinton Medium + 10% (*v*/*v*) Ethanol	2000
Ferulic acid	Mueller Hinton Medium + 10% (*v*/*v*) Ethanol	5000
Gallic acid	Mueller Hinton Medium	12,000
Humulene	Mueller Hinton Medium	2000
Myrcene	Mueller Hinton Medium	3000
Myricetin	Mueller Hinton Medium + 2% (*v*/*v*) Tween 80	200
*p*-Coumaric acid	Mueller Hinton Medium + 10% (*v*/*v*) Ethanol	5000
Syringic acid	Mueller Hinton Medium + 10% (*v*/*v*) Ethanol	6000
Thymol	Mueller Hinton Medium	4000
Vanillic acid	Mueller Hinton Medium + 2% (*v*/*v*) Tween 80	5000
Vanillin	Mueller Hinton Medium	10,000
Xanthohumol	Mueller Hinton Medium	50
β-Caryophyllene	Mueller Hinton Medium + 10% (*v*/*v*) Ethanol	3000

**Table 4:** Minimum Inhibitory Concentration (MIC) 50% and 90% of natural compounds for both Gram-negative (*E. coli*) and Gram-positive (*S. aureus*) bacteria.

		*E. coli*		*S. aureus*	
Natural Compound	Range (μg/mL)	MIC_50_ (μg/mL)	MIC_90_ (μg/mL)	MIC_50_ (μg/mL)	MIC_90_ (μg/mL)
3,4-Dihydroxybenzoic acid	0–3000	1500	3000	1500	3000
3-Hydroxybenzoic acid	0–1500	750	1500	750	1500
4-Hydroxybenzoic acid	0–1500	750	1500	750	1500
Caffeic acid	0–5000	2500	5000	2500	5000
Carvacrol	0–2150	65	130	180	280
Chlorogenic acid	0–2500	ND	ND	ND	ND
*p*-Coumaric acid	0–2500	625	1250	625	1250
Curcumine	0–25	ND	ND	ND	ND
Eugenol	0–1000	250	500	500	1000
β-Farnesene	0–1100	ND	ND	ND	ND
Ferulic acid	0–2500	625	1250	630	1250
Gallic acid	0–6000	6000	ND	1500	6000
Humulene	0–900	ND	ND	ND	ND
Myrcene	0–1450	ND	ND	ND	ND
Myricetin	0–100	ND	ND	ND	ND
Syringic acid	0–3000	750	1500	1500	3000
Thymol	0–2000	60	250	60	250
Vanillic acid	0–2500	625	1250	625	2500
Vanillin	0–5000	1250	2500	2500	5000
Xanthohumol	0–25	ND	ND	2	3
β-Caryophyllene	0–1500	ND	ND	ND	ND

ND: Not detected in the range of concentrations analyzed.

**Table 5:** Minimum Inhibitory Concentration (MIC) 50% and 90% of hop extracts for both Gram-negative (*E. coli*) and Gram-positive (*S. aureus*) bacteria.

		*E. coli*		*S. aureus*	
Hop Variety	Range (μg/mL)	MIC_50_ (μg/mL)	MIC_90_ (μg/mL)	MIC_50_ (μg/mL)	MIC_90_ (μg/mL)
Essential oils					
Nugget	0–900	ND	ND	900	ND
Cascade	0–900	ND	ND	10	30
Columbus	0–900	ND	ND	30	110
Fuggle	0–900	ND	ND	15	60
Magnum	0–900	ND	ND	30	115
Chinook	0–900	ND	ND	5	ND
Soft resins					
Nugget	0–800	50	200	15	50
Cascade	0–800	25	200	15	50
Columbus	0–800	25	200	15	50
Fuggle	0–800	50	200	15	25
Magnum	0–800	25	400	15	50
Chinook	0–800	12	50	25	100
Hard resins					
Nugget	0–800	750	ND	25	100
Cascade	0–800	100	ND	25	100
Columbus	0–800	800	ND	25	200
Fuggle	0–800	775	ND	50	200
Magnum	0–800	ND	ND	50	100
Chinook	0–800	ND	ND	50	100
Total resins					
Nugget	0–800	400	ND	50	100
Cascade	0–800	800	ND	15	50
Columbus	0–800	800	ND	15	50
Fuggle	0–800	800	ND	50	100
Magnum	0–800	ND	ND	15	50
Chinook	0–800	800	ND	25	100
Spent solids					
Nugget	0–300	ND	ND	5	ND
Cascade	0–300	ND	ND	450	ND
Columbus	0–300	ND	ND	10	ND
Fuggle	0–300	ND	ND	550	ND
Magnum	0–300	ND	ND	450	ND
Chinook	0–300	ND	ND	450	ND

ND: Not detected in the range of concentrations analyzed.

**Table 6:** Minimum Bactericidal Concentration (MBC) 99%, 99.9%, and 99.99% of natural compounds for both Gram-negative (*E. coli*) and Gram-positive (*S. aureus*) bacteria. Note: For each bacterium, if the highest MBC value of a compound could be determined within the range of concentrations analyzed, lower values were not evaluated and are indicated as not analyzed (NA).

		*E. coli*			*S. aureus*		
Natural Compound	Range (μg/mL)	MBC_99_ (μg/mL)	MBC_99.9_ (μg/mL)	MBC_99.99_ (μg/mL)	MBC_99_ (μg/mL)	MBC_99.9_ (μg/mL)	MBC_99.99_ (μg/mL)
3,4-Dihydroxybenzoic acid	0–3000	NA	NA	3000	NA	NA	3000
3-Hydroxybenzoic acid	0–1500	NA	NA	3000	NA	NA	3000
4-Hydroxybenzoic acid	0–1500	NA	NA	3000	NA	NA	3000
Caffeic acid	0–5000	NA	NA	5000	NA	NA	5000
Carvacrol	0–2150	NA	NA	125	NA	250	ND
Chlorogenic acid	0–2500	ND	ND	ND	ND	ND	ND
*p*-Coumaric acid	0–2500	NA	NA	1250	NA	2500	ND
Curcumine	0–25	ND	ND	ND	ND	ND	ND
Eugenol	0–1000	NA	NA	500	NA	1000	ND
β-Farnesene	0–1100	ND	ND	ND	ND	ND	ND
Ferulic acid	0–2500	NA	1250	ND	NA	NA	2500
Gallic acid	0–6000	ND	ND	ND	NA	6000	ND
Humulene	0–900	ND	ND	ND	ND	ND	ND
Myrcene	0–1450	ND	ND	ND	ND	ND	ND
Myricetin	0–100	ND	ND	ND	ND	ND	ND
Syringic acid	0–3000	NA	NA	3000	NA	3000	ND
Thymol	0–2000	NA	NA	250	NA	250	ND
Vanillic acid	0–2500	NA	NA	2500	NA	NA	2500
Vanillin	0–5000	NA	NA	5000	NA	5000	ND
Xanthohumol	0–25	ND	ND	ND	25	ND	ND
β-Caryophyllene	0–1500	ND	ND	ND	ND	ND	ND

ND: Not detected in the range of concentrations analyzed.

**Table 7:** Minimum Bactericidal Concentration (MBC) 99%, 99.9%, and 99.99% of hop extracts for Gram-positive (*S. aureus*) bacteria. Note: If the highest MBC value of a fraction type obtained from each hop variety could be determined within the range of concentrations analyzed, lower values were not evaluated and are indicated as not analyzed (NA).

*S. aureus*				
Hop	Range	MBC_99_	MBC_99.9_	MBC_99.99_
Variety	(μg/mL)	(μg/mL)	(μg/mL)	(μg/mL)
Essential oils				
Nugget	0–900	ND	ND	ND
Cascade	0–900	895	ND	ND
Columbus	0–900	445	ND	ND
Fuggle	0–900	NA	445	ND
Magnum	0–900	NA	900	ND
Chinook	0–900	ND	ND	ND
Soft resins				
Nugget	0–800	NA	NA	400
Cascade	0–800	NA	NA	200
Columbus	0–800	NA	NA	100
Fuggle	0–800	NA	NA	200
Magnum	0–800	NA	NA	200
Chinook	0–800	NA	NA	400
Hard resins				
Nugget	0–800	NA	NA	400
Cascade	0–800	NA	NA	800
Columbus	0–800	NA	NA	400
Fuggle	0–800	NA	NA	800
Magnum	0–800	NA	NA	400
Chinook	0–800	NA	NA	400
Total resins				
Nugget	0–800	NA	NA	400
Cascade	0–800	NA	NA	200
Columbus	0–800	NA	NA	200
Fuggle	0–800	NA	NA	400
Magnum	0–800	NA	NA	400
Chinook	0–800	NA	NA	400
Spent soilds				
Nugget	0–300	ND	ND	ND
Cascade	0–300	ND	ND	ND
Columbus	0–300	ND	ND	ND
Fuggle	0–300	ND	ND	ND
Magnum	0–300	ND	ND	ND
Chinook	0–300	ND	ND	ND

ND: Not detected in the range of concentrations analyzed.

## Data Availability

All data generated or analyzed during this study are included in this published article.

## Abbreviations

The following abbreviations are used in this article:

CECT	Colección española de cultivos tipo (Spanish type culture collection)
DSMZ	Deutsche sammlung von mikroorganismen und zellkulturen (German collection of microorganisms and cell cultures)
GAE	Gallic acid equivalents
MBC	Minimum Bactericidal Concentration
MBC_99_	Lowest concentration of the antimicrobial compound required to kill 99% of the viable cells
MBC_99.9_	Lowest concentration of the antimicrobial compound required to kill 99.9% of the viable cells
MBC_99.99_	Lowest concentration of the antimicrobial compound required to kill 99.99% of the viable cells
MIC	Minimum Inhibitory Concentration
MIC_50_	Lowest concentration of antimicrobial compound required to inhibit the growth of the 50% of the viable cells
MIC_90_	Lowest concentration of antimicrobial compound required to inhibit the growth of the 90% of the viable cells
MRB	Multidrug-resistant bacterial
NA	Not analyzed
ND	Not detected
PEP	Phosphoenolpyruvate
TPC	Total phenolic compounds

## References

[1] Fleming A. (1929). On the Antibacterial Action of Cultures of a *Penicillium*, with Special Reference to Their Use in the Isolation of *B. Influenzæ*. Br. J. Exp. Pathol..

[2] Barreiro C., García-Estrada C. (2019). Proteomics and *Penicillium chrysogenum*: Unveiling the Secrets behind Penicillin Production. J. Proteom..

[3] Ehrlich P., Hata S. (1910). Die Experimentelle Chemotherapie Der Spirillosen.

[4] Domagk G. (1935). Ein Beitrag Zur Chemotherapie Der Bakteriellen Infektionen. DMW—Dtsch. Med. Wochenschr..

[5] Abraham E.P., Chain E. (1988). An Enzyme from Bacteria Able to Destroy Penicillin. 1940. Rev. Infect. Dis..

[6] Kumarasamy K.K., Toleman M.A., Walsh T.R., Bagaria J., Butt F., Balakrishnan R., Chaudhary U., Doumith M., Giske C.G., Irfan S. (2010). Emergence of a New Antibiotic Resistance Mechanism in India, Pakistan, and the UK: A Molecular, Biological, and Epidemiological Study. Lancet Infect. Dis..

[7] Aminov R.I. (2010). A Brief History of the Antibiotic Era: Lessons Learned and Challenges for the Future. Front. Microbiol..

[8] Busch C., Noor S., Leischner C., Burkard M., Lauer U.M., Venturelli S. (2015). Anti-Proliferative Activity of Hop-Derived Prenylflavonoids against Human Cancer Cell Lines. Wien. Med. Wochenschr..

[9] Girisa S., Saikia Q., Bordoloi D., Banik K., Monisha J., Daimary U.D., Verma E., Ahn K.S., Kunnumakkara A.B. (2021). Xanthohumol from Hop: Hope for Cancer Prevention and Treatment. IUBMB Life.

[10] Kubeš J. (2022). Geography of World Hop Production 1990–2019. J. Am. Soc. Brew. Chem..

[11] Veiga B.A., Hamerski F., Clausen M.P., Errico M., de Paula Scheer A., Corazza M.L. (2021). Compressed Fluids Extraction Methods, Yields, Antioxidant Activities, Total Phenolics and Flavonoids Content for Brazilian Mantiqueira Hops. J. Supercrit. Fluids.

[12] Olšovská J., Kameník Z., Čejka P., Jurková M., Mikyška A. (2013). Ultra-High-Performance Liquid Chromatography Profiling Method for Chemical Screening of Proanthocyanidins in Czech Hops. Talanta.

[13] Sanz V., Torres M.D., López Vilariño J.M., Domínguez H. (2019). What Is New on the Hop Extraction?. Trends Food Sci. Technol..

[14] Bocquet L., Sahpaz S., Rivière C. (2018). An Overview of the Antimicrobial Properties of Hop.

[15] Paniagua-García A.I., Ruano-Rosa D., Díez-Antolínez R. (2023). Fractionation of High-Value Compounds from Hops Using an Optimised Sequential Extraction Procedure. Antioxidants.

[16] Almaguer C., Schönberger C., Gastl M., Arendt E.K., Becker T. (2014). *Humulus lupulus*—A Story That Begs to Be Told. A Review. J. Inst. Brew..

[17] Fahle A., Bereswill S., Heimesaat M.M. (2022). Antibacterial Effects of Biologically Active Ingredients in Hop Provide Promising Options to Fight Infections by Pathogens Including Multi-Drug Resistant Bacteria. Eur. J. Microbiol. Immunol..

[18] Lewis J.C., Alderton G., Carson J.F., Reynolds D.M., Maclay W.D. (1949). Lupulon and Humulon: Antibiotic Constituents of Hops. J. Clin. Investig..

[19] Etxeberria I., Garcia J., Ibáñez A., García-Moyano A., Paniagua-García A.I., Díaz Y., Díez-Antolínez R., Barrio A. (2025). Antimicrobial Activity of Lignin-Based Alkyd Coatings Containing Soft Hop Resins and Thymol. Coatings.

[20] Wongchum N., Dechakhamphu A. (2021). Xanthohumol Prolongs Lifespan and Decreases Stress-Induced Mortality in Drosophila Melanogaster. Comp. Biochem. Physiol. Part C Toxicol. Pharmacol..

[21] Natarajan P., Katta S., Andrei I., Babu Rao Ambati V., Leonida M., Haas G.J. (2008). Positive Antibacterial Co-Action between Hop (*Humulus lupulus*) Constituents and Selected Antibiotics. Phytomedicine.

[22] Sommella E., Pagano F., Salviati E., Chieppa M., Bertamino A., Manfra M., Sala M., Novellino E., Campiglia P. (2018). Chemical Profiling of Bioactive Constituents in Hop Cones and Pellets Extracts by Online Comprehensive Two-dimensional Liquid Chromatography with Tandem Mass Spectrometry and Direct Infusion Fourier Transform Ion Cyclotron Resonance Mass Spectrometry. J. Sep. Sci..

[23] Rúa J., del Valle P., de Arriaga D., Fernández-Álvarez L., García-Armesto M.R. (2019). Combination of Carvacrol and Thymol: Antimicrobial Activity against *Staphylococcus aureus* and Antioxidant Activity. Foodborne Pathog. Dis..

[24] Barry A.L., Lea & Febiger (1976). The Antimicrobic Susceptibility Test: Principles and Practices.

[25] Hintz T., Matthews K.K., Di R. (2015). The Use of Plant Antimicrobial Compounds for Food Preservation. Biomed. Res. Int..

[26] Barberis S., Quiroga H.G., Barcia C., Talia J.M., Debattista N. (2018). Natural Food Preservatives against Microorganisms. Food Safety and Preservation.

[27] Stan D., Enciu A.-M., Mateescu A.L., Ion A.C., Brezeanu A.C., Stan D., Tanase C. (2021). Natural Compounds with Antimicrobial and Antiviral Effect and Nanocarriers Used for Their Transportation. Front. Pharmacol..

[28] Saeed F., Afzaal M., Tufail T., Ahmad A., Var I., Uzunlu S. (2019). Use of Natural Antimicrobial Agents: A Safe Preservation Approach. Active Antimicrobial Food Packaging.

[29] Langezaal C.R., Chandra A., Scheffer J.J.C. (1992). Antimicrobial Screening of Essential Oils and Extracts of Some *Humulus lupulus* L. Cultivars. Pharm. Weekbl. Sci..

[30] Hrncic M.K., Španinger E., Košir I., Knez Ž., Bren U. (2019). Hop Compounds: Extraction Techniques, Chemical Analyses, Antioxidative, Antimicrobial, and Anticarcinogenic Effects. Nutrients.

[31] Jiang H., Zhong S., Schwarz P., Chen B., Rao J. (2023). Antifungal Activity, Mycotoxin Inhibitory Efficacy, and Mode of Action of Hop Essential Oil Nanoemulsion against *Fusarium graminearum*. Food Chem..

[32] Duarte P.F., do Nascimento L.H., Fischer B., Lohmann A.M., Bandiera V.J., Fernandes I.A., Magro J.D., Valduga E., Cansian R.L., Paroul N. (2023). Effect of Extraction Time on the Yield, Chemical Composition, and Antibacterial Activity of Hop Essential Oil against Lactic Acid Bacteria (*Lactobacillus brevis* and *Lactobacillus casei*) Beer Spoilage. Curr. Microbiol..

[33] Almeida A.d.R., Maciel M.V.d.O.B., Cardoso Gasparini Gandolpho B., Machado M.H., Teixeira G.L., Bertoldi F.C., Noronha C.M., Vitali L., Block J.M., Barreto P.L.M. (2021). Brazilian Grown Cascade Hop (*Humulus lupulus* L.): LC-ESI-MS-MS and GC-MS Analysis of Chemical Composition and Antioxidant Activity of Extracts and Essential Oils. J. Am. Soc. Brew. Chem..

[34] Lira M.H.P.d., Andrade Júnior F.P.d., Moraes G.F.Q., Macena G.d.S., Pereira F.d.O., Lima I.O. (2020). Antimicrobial Activity of Geraniol: An Integrative Review. J. Essent. Oil Res..

[35] Pontes E.K.U., Melo H.M., Nogueira J.W.A., Firmino N.C.S., de Carvalho M.G., Catunda Júnior F.E.A., Cavalcante T.T.A. (2019). Antibiofilm Activity of the Essential Oil of Citronella (*Cymbopogon nardus*) and Its Major Component, Geraniol, on the Bacterial Biofilms of Staphylococcus Aureus. Food Sci. Biotechnol..

[36] Fajdek-Bieda A., Pawlińska J., Wróblewska A., Łuś A. (2024). Evaluation of the Antimicrobial Activity of Geraniol and Selected Geraniol Transformation Products against Gram-Positive Bacteria. Molecules.

[37] Leite A.M., Lima E.d.O., Souza E.L.d., Diniz M.d.F.F.M., Trajano V.N., Medeiros I.A. (2007). de Inhibitory Effect of Beta-Pinene, Alpha-Pinene and Eugenol on the Growth of Potential Infectious Endocarditis Causing Gram-Positive Bacteria. Rev. Bras. Ciências Farm..

[38] Silva A.C.R.d., Lopes P.M., Azevedo M.M.B.d., Costa D.C.M., Alviano C.S., Alviano D.S. (2012). Biological Activities of A-Pinene and β-Pinene Enantiomers. Molecules.

[39] Teuber M., Schmalreck A.F. (1973). Membrane Leakage in *Bacillus subtilis* 168 Induced by the Hop Constituents Lupulone, Humulone, Isohumulone and Humulinic Acid. Arch. Mikrobiol..

[40] Kramer B., Thielmann J., Hickisch A., Muranyi P., Wunderlich J., Hauser C. (2015). Antimicrobial Activity of Hop Extracts against Foodborne Pathogens for Meat Applications. J. Appl. Microbiol..

[41] Sleha R., Radochova V., Mikyska A., Houska M., Bolehovska R., Janovska S., Pejchal J., Muckova L., Cermak P., Bostik P. (2021). Strong Antimicrobial Effects of Xanthohumol and Beta-Acids from Hops against *Clostridioides difficile* Infection in Vivo. Antibiotics.

[42] Yan Y.-F., Wu T.-L., Du S.-S., Wu Z.-R., Hu Y.-M., Zhang Z.-J., Zhao W.-B., Yang C.-J., Liu Y.-Q. (2021). The Antifungal Mechanism of Isoxanthohumol from *Humulus lupulus* Linn. Int. J. Mol. Sci..

[43] Bocquet L., Rivière C., Dermont C., Samaillie J., Hilbert J.-L., Halama P., Siah A., Sahpaz S. (2018). Antifungal Activity of Hop Extracts and Compounds against the Wheat Pathogen *Zymoseptoria Tritici*. Ind. Crops Prod..

[44] Rupasinghe H.P.V., Boulter-Bitzer J., Ahn T., Odumeru J.A. (2006). Vanillin Inhibits Pathogenic and Spoilage Microorganisms in Vitro and Aerobic Microbial Growth in Fresh-Cut Apples. Food Res. Int..

[45] Ngarmsak M., Delaquis P., Toivonen P., Ngarmsak T., Ooraikul B., Mazza G. (2006). Antimicrobial Activity of Vanillin against Spoilage Microorganisms in Stored Fresh-Cut Mangoes. J. Food Prot..

[46] Stroescu M., Stoica-Guzun A., Isopencu G., Jinga S.I., Parvulescu O., Dobre T., Vasilescu M. (2015). Chitosan-Vanillin Composites with Antimicrobial Properties. Food Hydrocoll..

[47] Rakchoy S., Suppakul P., Jinkarn T. (2009). Antimicrobial Effects of Vanillin Coated Solution for Coating Paperboard Intended for Packaging Bakery Products. Asian J. Food Agro-Ind..

[48] Guarda A., Rubilar J.F., Miltz J., Galotto M.J. (2011). The Antimicrobial Activity of Microencapsulated Thymol and Carvacrol. Int. J. Food Microbiol..

[49] Dietrich H., Pour Nikfardjam M.S. (2017). Influence of Phenolic Compounds and Tannins on Wine-Related Microorganisms. Biology of Microorganisms on Grapes, in Must and in Wine.

[50] Borges A., Ferreira C., Saavedra M.J., Simões M. (2013). Antibacterial Activity and Mode of Action of Ferulic and Gallic Acids against Pathogenic Bacteria. Microb. Drug Resist..

[51] Cueva C., Moreno-Arribas M.V., Martín-Álvarez P.J., Bills G., Vicente M.F., Basilio A., Rivas C.L., Requena T., Rodríguez J.M., Bartolomé B. (2010). Antimicrobial Activity of Phenolic Acids against Commensal, Probiotic and Pathogenic Bacteria. Res. Microbiol..

[52] Liu J., Du C., Beaman H.T., Monroe M.B.B. (2020). Characterization of Phenolic Acid Antimicrobial and Antioxidant Structure–Property Relationships. Pharmaceutics.

